# The impact of wavelengths of LED light-therapy on endothelial cells

**DOI:** 10.1038/s41598-017-11061-y

**Published:** 2017-09-06

**Authors:** Sabrina Rohringer, Wolfgang Holnthoner, Sidrah Chaudary, Paul Slezak, Eleni Priglinger, Martin Strassl, Karoline Pill, Severin Mühleder, Heinz Redl, Peter Dungel

**Affiliations:** 1 Ludwig Boltzmann Institute for Experimental and Clinical Traumatology, Donaueschingenstrasse 13, 1200 Vienna, Austria; 2 Austrian Cluster for Tissue Regeneration, Vienna, Austria; 3 Laser Consult Austria e.U., Salzburg, Austria; 4 Present Address: Max F. Perutz Laboratories, University of Vienna, Dr. Bohr-Gasse 9/3, 1030 Vienna, Austria

## Abstract

Low level light therapy receives increasing interest in the fields of tissue regeneration and wound healing. Several *in vivo* studies demonstrated the positive effects of LLLT on angiogenesis. This study aimed to investigate the underlying properties *in vitro* by comparing the effects of light therapy by light emitting diodes of different wavelengths on endothelial cells *in vitro*. Human umbilical vein endothelial cells were treated with either 475 nm, 516 nm or 635 nm light. Control cells were not illuminated. 2D proliferation was quantified by manual counting. HUVEC migration was analyzed by performing a 2D wound scratch assay and a 3D bead assay. The influence of LLLT on early vasculogenic events was determined in a 3D fibrin co-culture model with adipose-derived stem cells. Stimulation with both red and green pulsed LED light significantly increased HUVEC proliferation and 3D migration. Moreover, HUVEC showed increased 2D migration potential with green light stimulation. The treatment with blue light was ineffective. Several parameters showed that green light was even more potent to stimulate proliferation and migration of endothelial cells than clinically well-established red light therapy. Further studies have to focus on intracellular mechanisms induced by different wavelengths in order to optimize this promising therapy in tissue regeneration.

## Introduction

During the last few decades, numerous studies demonstrated the beneficial effects of low level light therapy (LLLT) for the treatment of various pathologies. Beside improvement of peripheral nerve regeneration^1^, reduction of inflammatory reactions^2^ and enhancement of bone formation^3^, the promotion of wound healing and angiogenesis represents an important field of application for LLLT^4, 5^.

Angiogenesis, the formation of new blood vessels out of pre-existing ones, is mediated by several growth factors, most prominently members of the vascular endothelial growth factors (VEGF) family. After secretion of these factors from the surrounding tissue endothelial cells, which line the inner walls of blood vessels, proliferate, migrate and form new capillary networks in sites of tissue damage. These small vessels are hyperpermeable, allowing the release of macromolecules for degrading the surrounding matrix which in turn facilitates the formation of more endothelial network structures^6^. It has been shown in several *in vivo* studies that vasculogenesis, the *de novo* formation of new capillary networks from endothelial progenitor cells, as well as angiogenesis can be triggered by mechanical or light stimulation^5, 7–9^.

Currently the main light sources for LLLT are lasers^10^. Ricci *et al*. investigated the effects of laser light on endothelial cell morphology, suggesting that LLLT influences the organization of endothelial cytoskeletons^11^. Schindl *et al*. observed a dose-dependent increase of human umbilical vein endothelial cell (HUVEC) proliferation by laser light^12^. Lasers, however, are associated with several disadvantages. Light emitting diodes (LED) can be effective alternative light sources, providing advantages like broad beam width and cost-efficiency. LED already moved into the focus of research and have recently been shown to be similarly effective. Park *et al*. showed that red, continuous LED light enhanced neovascularisation in a skin wound model in mice^9^. However, there is still controversy about the effectiveness of LED light versus lasers. So far, the majority of studies had been conducted with light in the red or infrared spectrum. There is increasing evidence, that shorter wavelengths can significantly support tissue regeneration processes. We could show that light can trigger the release of the important mediator nitric oxide (NO) bound to either mitochondrial proteins^13^ or hemoglobin^5^ and this release occurs in a wavelength-dependent manner with shorter wavelengths being more efficient. Adamskaya *et al*. compared the effects of different wavelengths in an excision model in rats and demonstrated that blue light was more potent to reduce wound size and enhance epithelialisation^14^. Dungel *et al*. showed that treatment with both red and blue LED light significantly enhanced angiogenesis and perfusion in a skin flap model in rats^8^.

A special feature in light therapy is the use of pulsed wave modes. Huang *et al*. reviewed the effect of pulsing in LLLT and concluded that currently there is some evidence that pulsed light indeed exerts effects that are different from those of continuous wave light^15^. Barolet *et al*. reported that specific pulsing patterns had a more favorable impact on the ability of fibroblasts to produce collagen *de novo* than comparative conditions of continuous wave^16^. Brondon *et al*. demonstrated that pulsing had a significantly greater stimulatory effect on cell proliferation and oxidative burst as compared to the continuous photoradiation group^17^.

So far, several studies revealed angiogenic and vasculogenic effects of lasers and LLLT *in vivo*, a systematic comparison between different wavelengths on the formation of vascular structures *in vitro* remained elusive. Therefore, in this study we aimed to evaluate the influence of pulsed LED light of three different wavelengths on endothelial cells. 2D as well as 3D proliferation and migration assays were performed. A well-established 3D fibrin gel co-culture model^18, 19^ with HUVEC and adipose-derived stem cells (ASC) was used to assess early vasculogenic events after one week of culture with continuous LLLT stimulation. In addition, protein expression levels in response to LLLT were determined.

## Results

### LLLT by red and green LED light increases the proliferation of HUVEC in a 2D culture environment while blue LED light decreases cell metabolic activity

**Figure 1:**
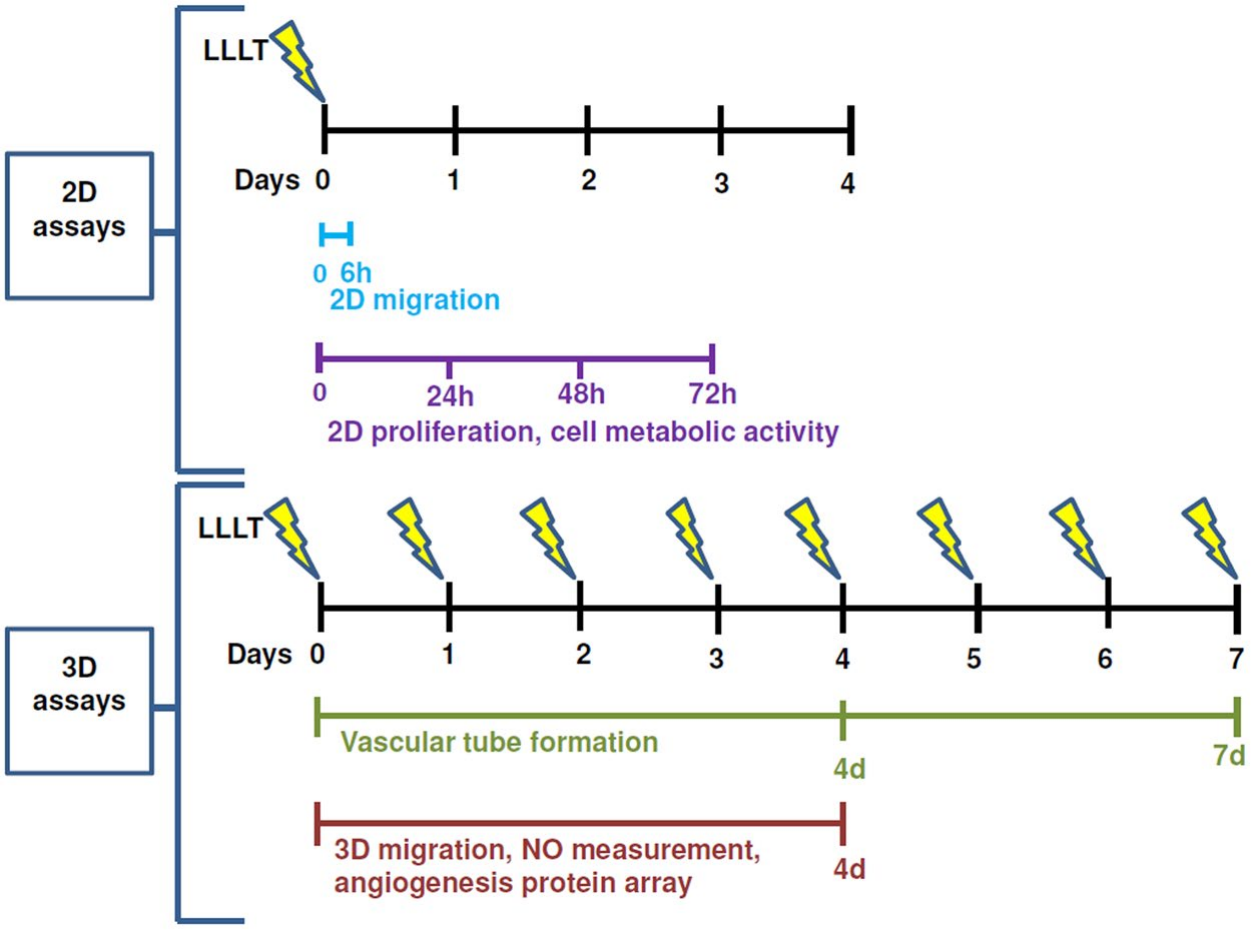
Experimental plan. Cells used for 2D experiments were stimulated on day 0 whereas cells embedded in 3D fibrin matrices were stimulation on day 0 and subsequently every 24 h until quantification was performed. HUVEC monolayers used for scratch assay were stimulated directly after performing the scratch and the migration was evaluated after 6 h. HUVEC for 2D proliferation experiments were stimulated after cells attached to the culture dish (approximately 2 hours after seeding) and counted every 24 h for 3 days. For 3D migration assays, NO measurement and angiogenesis protein array cells seeded to fibrin matrices were stimulated directly after polymerization of the fibrin clot and subsequently every 24 h for 4 days. Quantification of migrated cells was performed on day 4. 3D vascularization was determined by stimulating the cell-seeded fibrin clots directly after polymerization and every 24 h for one week. The quantification of 3D cell proliferation and vascularization was done after 4 and after 7 days.

**Figure 2:**
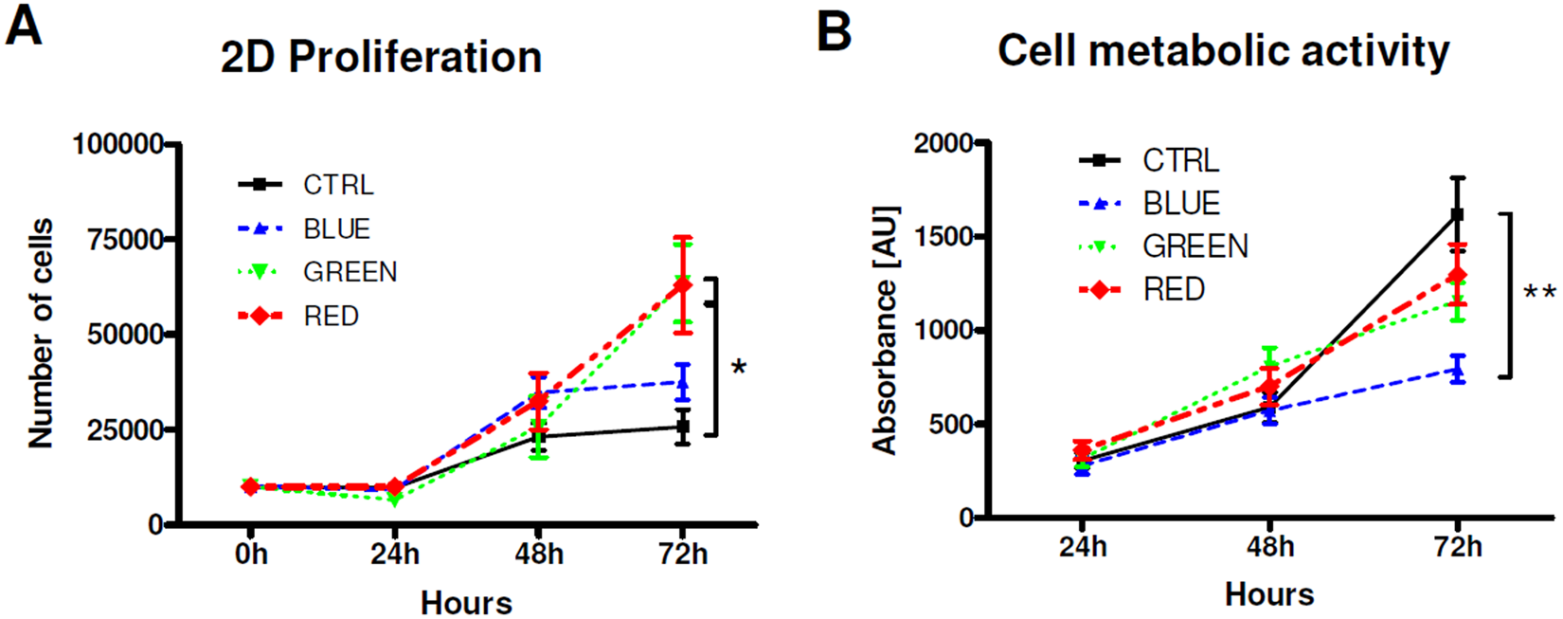
Effects of pulsed LED light on proliferation (**A**) and cell metabolic activity (**B**) of HUVEC cultured in a 2D cell culture model. Stimulation of HUVEC on day 0 with green and red light had no significant effect on metabolic activity while cell proliferation was significantly increased. In contrast, blue light significantly decreased metabolic activity and showed no effect on proliferation. *P < 0.05, **P < 0.01.

Figure 1 illustrates the study design. The effects of LLLT of different wavelengths on both proliferation and cell metabolic activity of endothelial cells were evaluated in HUVEC after initial light treatment at 24 h, 48 h and 72 h. Within the first 48 h after stimulation, cells proliferated similar in all stimulated and non-stimulated groups. At 24 h and 48 h there were no significant differences between any light treated group and untreated controls. After 72 hours, however, stimulation with green and red light significantly enhanced HUVEC proliferation. Mean values of cell counts were 146% higher in the green light group (P = 0.041) and 144% higher in the red light group (P = 0.043) compared to controls. Blue light treatment showed only a 45% increase compared to the control group (Fig. 2A). Regarding cell metabolic activity 72 h after light stimulation there was a decrease in all light treated groups compared to control, which reached statistical significance in the blue light group.

### HUVEC show significantly higher migration with green and red LED light stimulation

**Figure 3:**
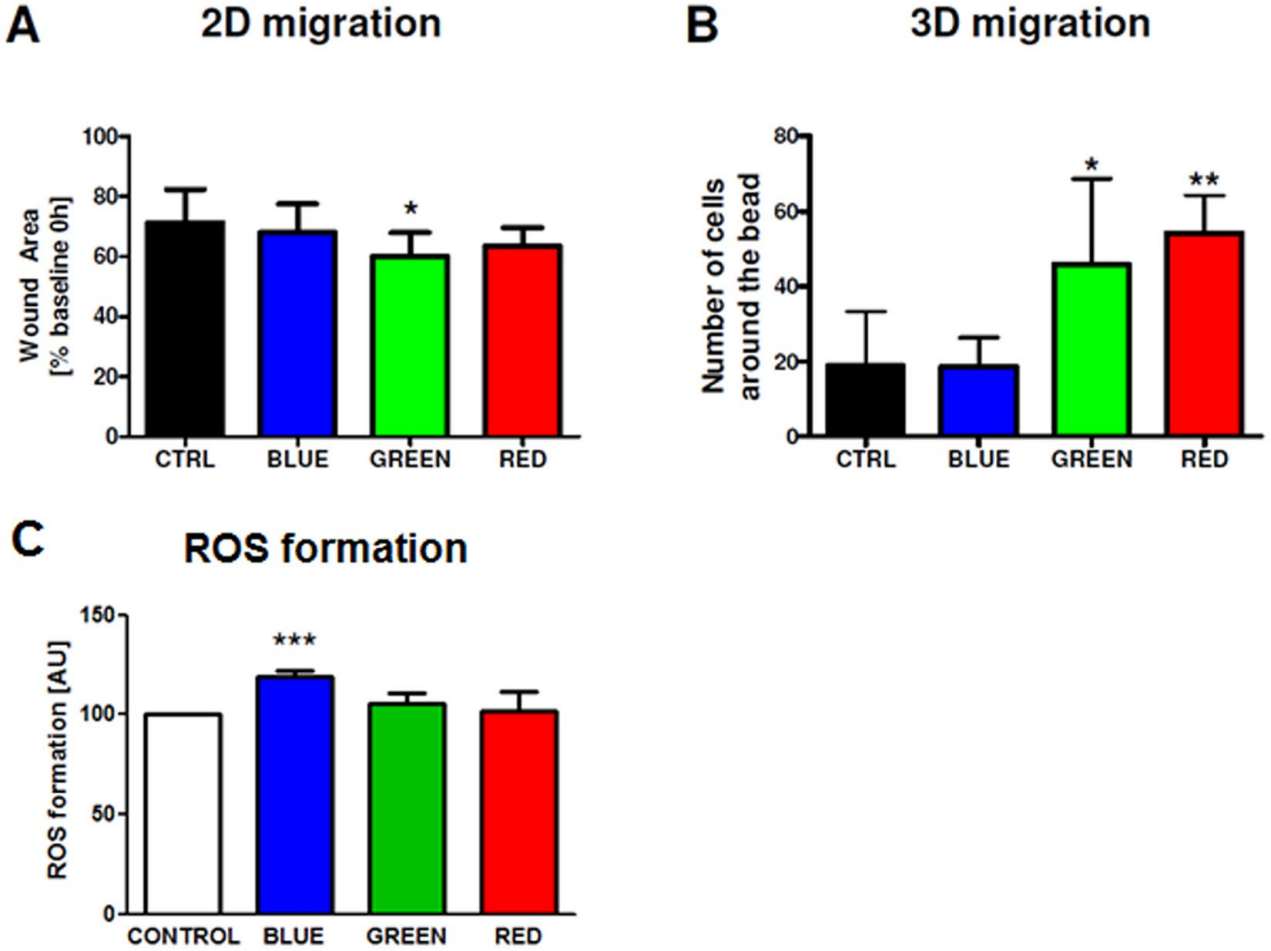
Effects of pulsed LED light on 2D- and 3D-migration *in vitro*. (**A**) Stimulation with light enhanced wound closure in scratch assays which reached significance in the green LED group. (**B**) In a 3D model of HUVEC-coated Cytodex 3 beads in fibrin matrices red and green light resulted in an increased migration of cells towards the fibrin gel while blue light was not effective. (**C**) Effects of pulsed LED light on ROS formation. Only blue light induced a significant rise in ROS. *P < 0.05, **P < 0.01, ***P < 0.001.

In order to analyze pro-migratory effects of LLLT on HUVEC *in vitro*, 2D wound scratch assays as well as 3D migration assays were performed. Green light stimulation significantly enhanced HUVEC 2D migration leading to faster reduction of the wound scratch area determined at 6 h after LLLT compared control (Fig. 3A). While in the untreated control group wound area was reduced within 6 h by only 28.7 ± 11.1%, there was a reduction of 32.0 ± 9.6% in the blue light group, 36.6 ± 6.3% in the red light group and 40.1 ± 8.0% in the green light treated group which was statistically significant compared to control (P = 0.046).

Additionally, migration was also investigated in a 3D cell culture model. The quantification of cells migrating away from Cytodex beads into the surrounding fibrin gel matrix showed that both red and green light had significant influence on 3D migration after 4 days (Fig. 3B). While in the control group 19.8 ± 16.2 cells were migrating away from the beads, this number significantly increased in the green light group to 47.8 ± 19.9 cells (P = 0.047) and to 53.2 ± 7.1 cells in the red group (P = 0.0041). Blue light showed no effect and was in the same range as the control group.

### Effects of LLLT on endothelial cells embedded in 3D fibrin matrices

**Figure 4:**
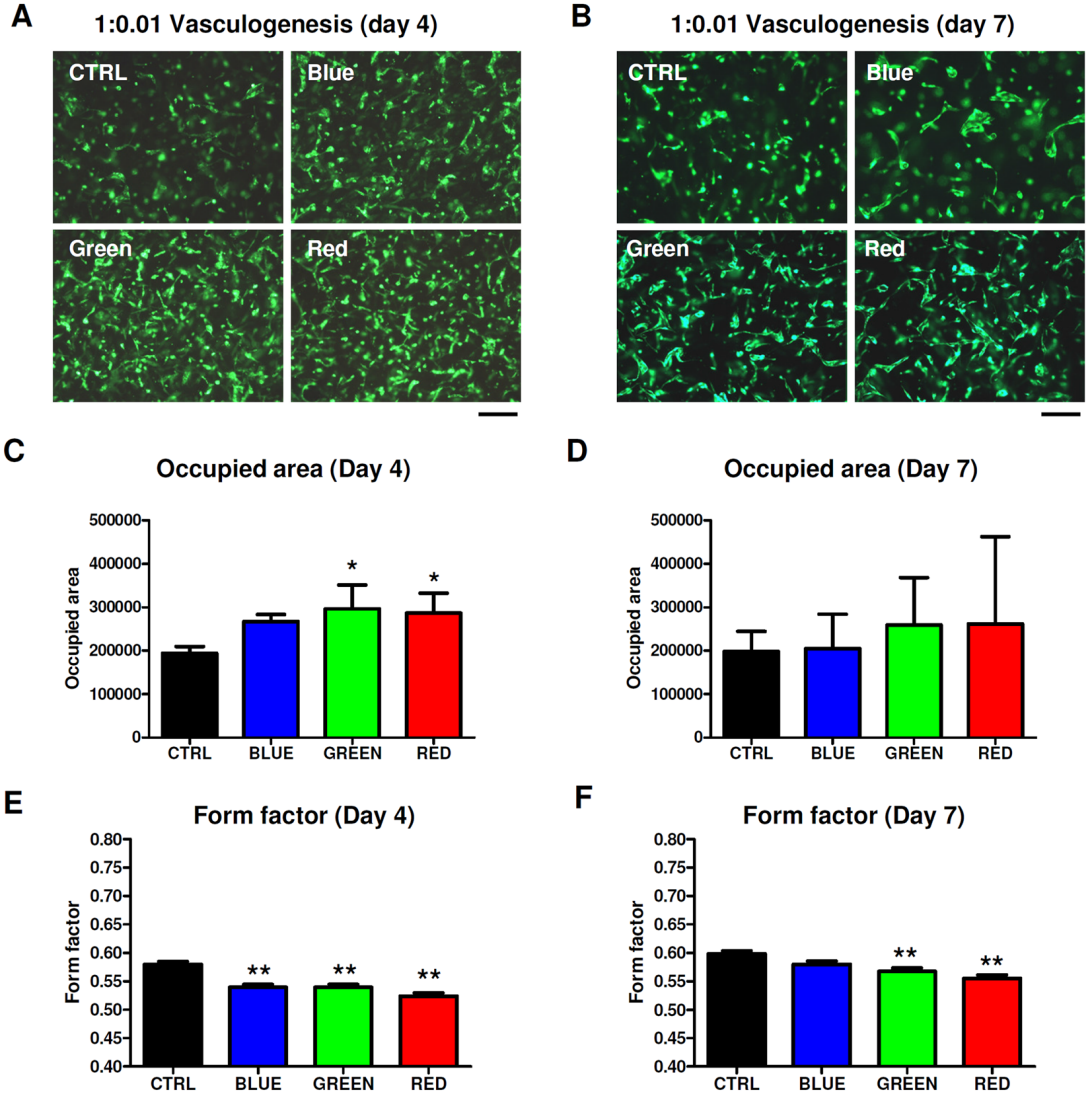
Effects of pulsed LED light on 3D cell proliferation and vasculogenesis in 1:0.01 co-cultures of HUVEC with ASC. (**A**,**B**) Representative images of LED stimulated fibrin clots containing GFP-HUVEC/ASC co-cultures in a ratio of 1:0.01 after 4 days (**A**) and 7 days (**B**) of culture. (**C**) An increase of the area occupied by cells was determined in all light treated groups which reached significance with red and green light. (**D**) A trend to enhanced proliferation determined as occupied area was still observable after 7 days with red light being more potent. This effect, however, was not significant € The form factor of GFP-HUVEC was significantly reduced in all LED light stimulated groups, indicating increased cell stretching necessary to form cell-cell interactions. (**F**) After 7 days cell elongation was detectable in cells treated with green and red light. (**G**) Quantification of cellular junctions in 1:1 HUVEC/ASC co-cultures after one week showed a trend to enhanced network formation with red light treatment and a significant influence of green light. *P < 0.05, **P < 0.01.

The effects of LED light of different wavelengths on endothelial cells placed on an extracellular matrix were tested using the Matrigel assay. Neither wavelength did have a significant effect on the formation of primitive endothelial networks (Supp. Figure 1). Since this assay is restricted to vasculogenic influences within 24 h we proceeded with our well-established 3D co-culture model of GFP-HUVEC and human ASC in a fibrin matrix (1:0.01 HUVEC/ASC seeding ratio). Figure 4 shows representative images of stimulated 3D co-cultures after 4 days (Fig. 4A) and on day 7 (Fig. 4B). At the 1:0.01 ratio no mature network formation was detected within 7 days. However, cells in the LED-stimulated groups showed increased cell elongation indicating enhanced activity to form cell-cell interactions. Quantification of the occupied area confirmed also in this co-culture model that stimulation with green and red light increased GFP-HUVEC proliferation (Fig. 4C). The area occupied by cells was significantly enhanced by red (~40%) and green (~45%) light treatment. The quantification in Fig. 4E represents form factor changes of GFP-HUVEC in HUVEC/ASC co-cultures (1:0.01) after 4 days. Here, a form factor reduction of 10% in all LED light treated groups was observed. After 7 days of culture, the was still a trend in the green and red light treated group to a larger cell-occupied area compared to control (Fig. 4D). Accordingly, the form factor was still significantly decreased by approximately 15% in the red and green light treated group (Fig. 4F). Using mCherry-labelled ASC and GFP-labelled HUVEC in a 1:1 ratio we see the two cell types interacting (Sup. Figure 2A,B). The formation of cell-cell junctions in a 1:1 ratio of ASC:EC after one week was verified by immunostaining with antiCD31 antibodies (Supp. Figure 3A,B).

### LLLT elevates NO production of HUVEC/ASC co-cultures

**Figure 5:**
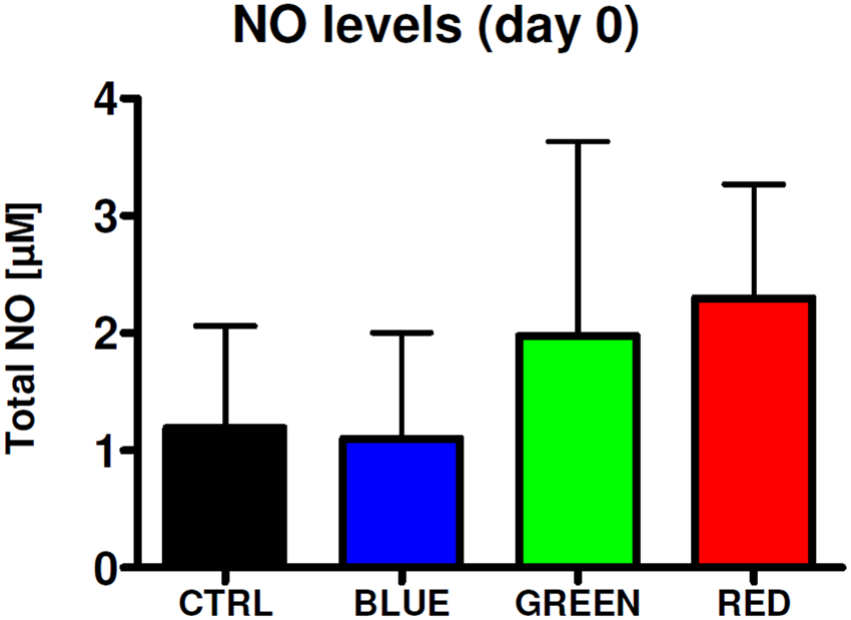
Effects of pulsed LED light on NO levels in the supernatant of HUVEC/ASC co-cultures. Cells were illuminated at different wavelengths and supernatants immediately drawn and frozen for NO determination via chemiluminescence-based analysis.

NO levels were determined after light treatment in the cell culture supernatant. There was a trend to increased NO formation in the red and green light treated group compared to control immediately after light treatment (Fig. 5). No differences were detected after 4 days.

### LLLT influences the protein expression in HUVEC/ASC co-cultures

To evaluate if LLLT has effects on the expression of angiogenesis-related proteins, 55 different angiogenesis-related cytokines were analyzed in the supernatants of HUVEC/ASC co-cultures (1:0.01) (Table 1). Dependent on the wavelength various factors were influenced by light. Blue light upregulated dipeptidylpeptidase IV (DPPIV) by 90%, and neuregulin1-b1 (NRG1-b1) by approximately 50%. Moreover, placenta growth factor (PIGF) levels were 80% higher after blue light stimulation. Green light treatment led to an increase of hepatocyte growth factor (HGF) by 60% and an enhancement of NRG1-b1 by 120%. Furthermore, serpin F1 was upregulated by 380%. HGF levels were also higher after red light stimulation (almost 100% increase). In addition, the chemokine CXCL16 was 1.6 fold increased after red light treatment. Interestingly, the prominent factors VEGF and TGF-β were not markedly changed.

**Table 1:** Effects of pulsed LED light on protein expression levels of HUVEC/ASC.

	RED	GREEN	BLUE
Activin A	1,29 ± 0,08	1,14 ± 0,02	0,96 ± 0,08
ADAMTS-1	1,19 ± 0,06	1,27 ± 0,09	1,20 ± 0,06
Angiogenin	1,25 ± 0,00	0,87 ± 0,02	1,16 ± 0,07
Angiopoietin-1	0,95 ± 0,01	0,61 ± 0,02	1,00 ± 0,02
Angiopoietin-2	1,16 ± 0,04	0,61 ± 0,09	1,02 ± 0,01
Angiostatin	1,33 ± 0,07	0,81 ± 0,04	1,25 ± 0,06
Amphiregulin	1,29 ± 0,05	0,70 ± 0,01	1,35 ± 0,05
Artemin	1,42 ± 0,01	0,76 ± 0,01	1,14 ± 0,01
Tissue Factor	1,19 ± 0,06	0,66 ± 0,04	1,11 ± 0,05
CXCL16	1,65 ± 0,03	1,00 ± 0,01	1,31 ± 0,01
DPPIV	1,16 ± 0,16	1,09 ± 0,10	1,94 ± 0,15
EGF	1,24 ± 0,01	0,69 ± 0,00	1,05 ± 0,00
EG-VEGF	1,33 ± 0,02	0,73 ± 0,10	1,13 ± 0,03
CD105	1,35 ± 0,08	0,66 ± 0,03	1,13 ± 0,10
Endostatin/Collagen XVIII	1,36 ± 0,02	0,83 ± 0,00	0,66 ± 0,02
Endothelin-1	1,29 ± 0,04	0,72 ± 0,01	1,02 ± 0,05
FGF-1	1,31 ± 0,12	0,58 ± 0,03	1,09 ± 0,00
FGF-2	1,45 ± 0,10	0,87 ± 0,04	1,20 ± 0,05
FGF-4	1,24 ± 0,01	0,66 ± 0,03	1,07 ± 0,05
FGF-7	1,16 ± 0,02	0,68 ± 0,08	0,90 ± 0,01
GDNF	1,19 ± 0,04	0,74 ± 0,09	0,85 ± 0,00
GM-CSF	1,41 ± 0,09	0,84 ± 0,01	1,06 ± 0,03
HB-EGF	1,19 ± 0,14	0,94 ± 0,02	1,10 ± 0,10
HGF	2,01 ± 0,08	1,65 ± 0,13	1,36 ± 0,10
IGFBP-1	1,00 ± 0,01	0,73 ± 0,05	0,75 ± 0,04
IGFBP-2	1,40 ± 0,04	0,93 ± 0,02	1,13 ± 0,00
IGFBP-3	1,15 ± 0,02	0,90 ± 0,06	0,67 ± 0,03
IL-1β	0,91 ± 0,03	1,92 ± 1,28	1,09 ± 0,10
IL-8	1,27 ± 0,05	0,90 ± 0,02	1,07 ± 0,00
LAP (TGF-β1)	1,28 ± 0,13	0,94 ± 0,09	1,00 ± 0,06
Leptin	1,17 ± 0,00	0,87 ± 0,02	1,22 ± 0,00
MCP-1	1,26 ± 0,01	0,88 ± 0,01	0,93 ± 0,02
MIP-1α	1,18 ± 0,05	0,84 ± 0,04	0,87 ± 0,02
MMP-8	1,14 ± 0,02	0,90 ± 0,00	0,88 ± 0,06
MMP-9	1,35 ± 0,13	0,94 ± 0,11	1,11 ± ± 0,06
NRG1-β1	0,95 ± 0,05	2,17 ± 1,59	1,53 ± 0,42
Pentraxin 3 (PTX3)	1,40 ± 0,01	0,97 ± 0,02	0,99 ± 0,00
PD-ECGF	1,26 ± 0,07	0,96 ± 0,07	0,97 ± 0,02
PDGF-AA	1,19 ± 0,05	0,84 ± 0,08	0,86 ± 0,01
PDGF-AB/PDGF-BB	1,12 ± 0,15	0,98 ± 0,02	0,96 ± 0,03
Persephin	0,87 ± 0,04	0,78 ± 0,00	0,94 ± 0,01
Platelet Factor 4 (PF4)	1,07 ± 0,07	0,92 ± 0,02	0,96 ± 0,04
PIGF	1,08 ± 0,01	1,24 ± 0,05	1,73 ± 0,08
Prolactin	1,01 ± 0,06	0,90 ± 0,08	0,98 ± 0,02
Serpin B5	1,39 ± 0,02	0,92 ± 0,03	1,07 ± 0,05
Serpin E1	1,39 ± 0,03	1,00 ± 0,03	1,03 ± 0,04
Serpin F1	1,04 ± 0,17	3,67 ± 2,49	1,06 ± 0,01
TIMP-1	1,24 ± 0,03	0,91 ± 0,00	1,05 ± 0,01
TIMP-4	1,01 ± 0,08	0,90 ± 0,00	1,19 ± 0,13
Thrombospondin-1	2,40 ± 0,02	0,77 ± 0,13	1,08 ± 0,06
Thrombospondin-2	1,24 ± 0,08	0,96 ± 0,06	1,14 ± 0,03
uPA	1,21 ± 0,05	0,85 ± 0,05	0,84 ± 0,00
Vasohibin	1,05 ± 0,05	0,79 ± 0,03	0,96 ± 0,01
VEGF	0,88 ± 0,00	0,72 ± 0,01	0,80 ± 0,13
VEGF-C	1,18 ± 0,05	0,87 ± 0,08	1,09 ± 0,02

After stimulation of HUVEC/ASC fibrin co-cultures with LLLT for 4 days every 24 h, DDPIV, NRG1-b1 and PIGF were upregulated with blue light. HGF levels were increased after green as well as red LLLT. Similar to blue LLLT, clots stimulated with green light showed enhanced levels of NRG1-b1, but also serpin F1. The expression of CXCL16 was increased after red light stimulation. Experiments were performed with pooled supernatants from three donors. Values represent means ± standard deviation from two technical replicates.

## Discussion

Low level light therapy (LLLT) is a promising and fast expanding physical approach in various medical fields, especially to reduce pain and inflammation and support tissue regeneration^20^. Numerous *in vivo* studies indicated a positive effect of LLLT by laser light on wound healing associated with enhanced angiogenesis^4, 21^. Endothelial cells are vital in wound healing and play a key role in angiogenesis. The direct effect of light on endothelial cells using lasers in the red spectrum has already been reported. Investigating the impact of laser light treatment on endothelial cells *in vitro* Schindl *et al*. showed positive effects of LLLT with 670 nm on HUVEC proliferation^12^. Also Chen *et al*. demonstrated the stimulatory effect of LLLT on HUVEC proliferation^22^ and concluded that laser irradiation increases endothelial cell proliferation, migration, and eNOS gene expression possibly by signalling via phosphoinositid-3 kinases. Szymanska *et al*. reported that increased proliferation of HUVEC mediated by light stimulation is mediated by an increase of VEGF and transforming growth factor beta (TGF-β) levels^23^. However, there is still controversy whether LED light can be an efficient alternative light source to lasers^24^. In a skin flap model in rats Dungel *et al*. confirmed that red LED light induces pro-angiogenic effects, but found that also blue light can significantly increase angiogenesis^8^. The direct impact of LED light of different wavelengths on endothelial cells was not investigated so far. Thus, in the present study we compared the impact of LLLT by pulsed LED light of 475 nm, 516 nm and 635 nm on regenerative processes in HUVEC. Light treatment of HUVEC in standard 2D cultures led to significant increased proliferation in the green and red light treated group 72 h after LLLT while there was only a trendwise increase in the blue light treated group. At this time point metabolic activity was decreased in all light treated groups which reached statistical significance only in the blue light group. It has been reported that when using biophysical stimuli, data of proliferation and metabolic activity are not directly correlated. A study by Balzer *et al*. showed that an increase in cell activity by a resazurin-based assay was associated with a decrease in Hoechst-stained nuclei counts^25^.

Quent *et al*. reported on the discrepancies between metabolic activity and DNA content and concluded that metabolic assays may not accurately reflect cellular proliferation rates due to a miscorrelation of metabolic activity and cell number^26^. Interestingly, although in this model light was only applied once, there were still significant effects observed 72 hours after stimulation. This is in line with recent data from our lab which showed that treatment by extracorporeal shock waves also had effects on EC and ASC proliferation several days after one-time treatment^7, 27^. These data may indicate that long-term cellular effects can be initiated by light therapy.

LLLT can either act directly on cells but also could initiate the release of specific mediators. As a first step to evaluate the secretom we analysed light-induced ROS formation. Low levels of ROS are important for signaling but higher doses can induce detrimental effects. We showed that, in contrast to red and green light, blue light led to a significant formation of ROS, which might correlate with the negative effects of blue light on proliferation and migration.

Vasculogenesis involves complex steps of proliferation and migration of endothelial cells and the maturation of primitive vascular structures. We therefore performed 2D as well as 3D migration experiments with human endothelial cells. The reduction of artificially created 2D scratch wounds was trendwise reduced by red LED light and reached significance with green LED light treatment. The positive effect of red light is in line with the report of Teuschl *et al*. showing in scratch wound models that LLLT with red LED light increased myoblast, fibroblast and keratinocyte migration *in vitro* while blue LED light was ineffective^28^. Similar effects were seen when quantifying the migration of HUVEC in a 3D assay with enhanced migration in the green and red light stimulated groups while blue light was not effective. Fushimi *et al*. indicated similar effects in human keratinocytes and showed that red and green LED light stimulation enhances wound healing^29^.

As endothelial cells are important for angiogenesis we aimed to investigate light-induced network formation in two separate *in vitro* models which mimick early events in angiogenic and vasculogenic processes. In a Matrigel assay no effects of either wavelength were detected. However, this assay only reflects early vasculogenic events within approx. 24 hrs. Amongst other disadvantages, Matrigel contains an undefined mixture of pro-angiogenic cytokines derived from a mouse brain tumor. Thus, we also investigated the effects of LLLT in an *in vitro* 3D co-culture model developed in our lab. Using this model we have recently shown that mechanical stimulation with shockwaves enhances HUVEC vascular tube formation^7^. In order to evaluate if light of different wavelengths may have similar stimulating effects, fibrin clots containing HUVEC and ASC at a ratio of 1:0.01 were stimulated with LLLT and the precursors of vascularization was quantified over 7 days. Since in previous studies it has been shown that a 1:1 seeding ratio usually leads to mature capillary network within one week even without any stimulation, a seeding ratio of 1:0.01 was chosen in order to observe the stimulating effects of LLLT on vasculogenic processes. At 1:0.01 ratio there was no network formation within 7 days of culture. However, it was possible to detect and quantify single-cell specific changes in morphology. Light treatment at all used wavelengths led to significant stretching and elongation of HUVEC on day 4. This data is supported by Ricci *et al*. who investigated the effects of laser light on endothelial cell morphology and suggested that LLLT influences the organization of endothelial cytoskeletons^11^. In this model also proliferation was significantly enhanced by red and green light determined by analysis of the cell-occupied area which is in accordance with the data obtained from the 2D proliferation assay. The positive trend of increased proliferation continued until day 7. Stretching and elongation of cells was still significantly enhanced by green and red light on day 7. In the present study positive effects on regenerative processes were demonstrated for green and red light while blue light was not effective. The discrepancy between the positive effects of blue LED light *in vivo* ^5^ and inefficiency *in vitro* as shown in this study might indicate different underlying mechanisms. Dungel *et al*. showed that blue light is most efficient to release the important mediator nitric oxide (NO) bound to mitochondrial complexes^13^. In *in vivo* situations of ischemic wound healing NO is reduced from nitrite via oxygen-independent nitrite reductases. Thus, NO-mediated effects induced by blue light may play an important role in *in vivo* settings. The formation of NO was analysed in cell culture supernatants immediately after illumination as well as on day 4. Although not significant, both red and green light induced higher NO levels, which might be associated with the positive effects of these wavelengths on proliferation and migration. The finding that NO and ROS levels are indirectly correlated might once more point to different pathways activated by different wavelengths. Red light has also been reported to act via photobiomodulation of complexes in the respiratory chain of mitochondria, mainly cytochrome c oxidase. The role of green light in these processes has to be further elucidated. By observing expressed protein levels after LLLT, we found that pro-angiogenic factors such as HGF, NRG1-b1, PIGF, CXCL16 and DPPIV as well as the anti-angiogenic factor serpin F1 were upregulated after LLLT. Ieda *et al*. showed that HGF improves the recovery of hind limb ischemia by promoting angiogenesis^30^. Further, endothelial-derived NRG has an important function in ischemia-induced angiogenesis and arteriogenesis^31^. DPPIV serves as a mediator of angiogenesis and tissue remodelling and serves as a necessary protein involved in the proliferation of smooth muscle cells^32^. Interestingly, the expression of the analysed proteins did not correlate very well with the observed effects on migration and wound healing. The anti-angiogenic factor Serpin F1 was upregulated only in the green light group, while blue light that was ineffective upregulated only pro-angiogenic factors. However, the co-culture model does not necessarily reflect *in vivo* angiogenesis but rather vasculogenic events. The formation of vascular structures includes remodeling processes and a balance of pro- and anti-angiogenic factors. Our *in vitro* findings suggest that an interplay of pro-angiogenic, remodelling, but also anti-angiogenic factors such as serpin F1 could be the driving factors of LLLT-mediated wound healing and angiogenesis *in vivo*.

In summary, LLLT by red and green LED light increased migration and proliferation of endothelial cells consistently in several independent models. Especially the positive effects of green light are novel and have to be tested in further studies. The difference in the effects of blue light *in vivo* compared to *in vitro* raises interesting issues on the mechanisms of light therapy. Certainly *in vivo* situations are much more complex with many components coming into play. In our previous reports in *in vivo* models, in which blue light was effective, wound healing has been disturbed by ischemia. In this setting oxygen-independent nitrite synthases are active to bioactivate nitrite to NO and light can influence the activity of these enzymes^33^. Blue light is also most efficient to release NO which is bound to mitochondrial and other heme proteins^5, 13^. NO has been shown to play a key role in wound healing. Also light-induced NO-dependent vasodilation takes place *in vivo* ^34^. All these mechanisms do not occur in *in vitro* systems. Therefore it is not remarkable that there are differences between *in vitro* and *in vivo*. However, *in vitro* studies are valuable and necessary to investigate the role and effects of selected cell types. Thus, although insight into possible mechanisms was gained in the last few years by performing *in vitro* investigations^35^, the influence of different wavelengths may still hold great potential.

## Methods

### Cells

All experimental protocols were approved by the Ethics Committee of the Land Upper Austria. The informed consent was obtained from all subjects. The methods were carried out in accordance with the approved guidelines.

HUVEC were either purchased from Lonza (C2519-A or C2856; Lonza, Basel, Switzerland) or isolated from fresh umbilical veins as described before^36^. Green fluorescent protein (GFP) expressing HUVEC were purchased from Olaf pharmaceuticals (GFP; Olaf pharmaceuticals, Worcester, USA). The collection of human adipose tissue was approved by the local ethical board with patient’s consent. Subcutaneous adipose tissue was obtained during routine outpatient liposuction procedures under local tumescence anaesthesia. Human ASC were isolated from liposuction material as described before^37, 38^. HUVEC, GFP-HUVEC and ASC were cultured in endothelial growth medium (EGM-2; Lonza) supplemented with 5% fetal calf serum (FCS; GE Healthcare, Chalfont St Giles, UK) at 37 °C with 5% CO_2_. Endothelial cells were maintained in cell culture flasks (TPP, Trasadingen, Switzerland) coated with 2 µg/ml bovine fibronectin (Sigma-Aldrich, St. Louis, USA) whereas ASC were cultured on uncoated plastic surfaces (TPP). For experiments cells were seeded in 24-well plates.

### LLLT stimulation

LED lamps for light therapy were provided by Repuls Lichtmedizintechnik GmbH, Austria. Cells were treated with pulsed LED light of either 475 nm (blue), 516 nm (green), 635 nm (red) or remained unstimulated (control). All LED devices had a peak irradiance intensity of 80 mW/cm^2^ which was measured with a USB 2000 spectrometer (Ocean Optics, FL, USA). Given the pulse rate of 50% and a repetition frequency of 2.5 Hz an average irradiance intensity of 40 mW/cm^2^ was reached. The daily dose provided was 24 J/cm^2^. Illumination was performed at room temperature for 10 min at a distance of 2 cm from the cells. For 2D experiments, cells were stimulated on day 0 only whereas for 3D experiments stimulation was performed every 24 hours (Fig. 1).

### Proliferation and MTT assays

To assess the effects of light treatment on HUVEC proliferation, 10^4^ HUVEC were seeded to 24-well plates for each condition per time point and grown until 20% confluence. Cells were stimulated by LLLT on day 0 and further cultivated for up to 72 hours. Every day duplicates of cells were enzymatically detached with trypsin (Sigma-Aldrich) and counted with a Neubauer counting chamber (VWR, Darmstadt, Germany).

To assess the effects of light treatment on HUVEC cell metabolic activity assay, HUVEC were as described above. However, instead of detaching, every day duplicates of cells subjected to MTT assays. Briefly, the supernatants were removed and cells were washed three times with PBS. MTT reagent (Sigma) was dissolved in 1× PBS (5 g/L). Before adding to the cells, this stock solution was diluted with the respective growth medium to 3.25 g/L and the plate was incubated for 1 hour at 37 °C. The supernatant was removed and formazan crystals were dissolved using dimethyl sulfoxide (Sigma). The plate was incubated on a shaker in the dark at room temperature for 20 min and absorbance was measured at 540 nm with 650 nm as reference.

### 2D migration- Scratch assay

Scratch assays were performed as described previously^7^. Briefly, 10^4^ HUVEC were seeded to wells of a 24-well plate and grown until 100% confluence. To investigate migration in a 2D monolayer, an artificial wound was created by scraping the cells off the well with a 1000 µl pipet tip (Greiner bio one, Kremsmünster, Austria) within a certain area. Scraped cells were removed by washing the monolayer twice with 1x phosphate buffer saline (PBS). 1 ml of EGM-2 medium was added to each well and LLLT was performed immediately. Images of the scratched wounds were taken directly after stimulation (0 h) and after a 6 hours incubation time at 37 °C. Three images per scratch were taken to cover the whole scratched area of each well. In order to quantify the cell migration into the artificial wound, the cell-free area was quantified with ImageJ using the area measurement tool.

### 3D migration- bead assay

To analyze the effects of LLLT on 3D migration of HUVEC, cell-coated Cytodex 3 beads (GE Healthcare) were incorporated into 3D fibrin matrices and the migration of cells from the beads into the fibrin matrix was quantified. Therefore, cells at a seeding concentration of approximately 100 cells per bead (2 × 10^5^ beads/ml) were incubated with beads in EGM-2 for 4 hours at 37 °C with occasional shaking every 20 min to ensure an even cell coating of the beads. The beads were then incubated overnight. The next day, 5 µl bead suspension (approximately 100 beads) was added to 5 µl of fibrinogen (100 mg/ml; Baxter, Vienna, Austria) and 90 µl EGM-2. The fibrinogen solution containing the cells was then mixed with 100 µl thrombin (0.4 U/ml, Baxter, Vienna, Austria) resulting in fibrin gels with a final fibrin concentration of 2.5 mg/ml and a total volume of 200 µl. These matrices were prepared on round coverglasses with 15 mm diameter (VWR, Radnor, USA) in 24 well plates. After polymerization at 37 °C for 20 min EGM-2 was added to the wells. Gels on the coverglasses were stimulated 2 hours after polymerization of the fibrin gel and subsequently every 24 hours until measurements and quantifications were conducted. The fibrin gels were stimulated every day for 4 consecutive days. The gels were fixed with 4% paraformaldehyde (PFA, Sigma-Aldrich) for 6 hours at 4 °C while shaking and cell nuclei were stained with 4′,6-diamidin-2-phenylindol (DAPI) subsequently for 4 hours at 4 °C. The amount of migrated cells away from beads to the surrounding fibrin matrix was quantified by counting DAPI-positive cells in a determined region around the beads.

### HUVEC/ASC co-cultures in 3D fibrin matrices

3D co-cultures of GFP-HUVEC and human ASC in fibrin gels were prepared as described previously^7, 18, 19^. In brief, both cell types were enzymatically detached with trypsin, counted, adjusted to a volume of 95 µl and mixed with 5 µl of 100 mg/ml fibrinogen (Baxter, Vienna, Austria) to a total volume of 100 µl. GFP-HUVEC and ASC were either used in a 1:1 ratio (10^5^ cells per cell type in each gel) or in a 1:0.01 ratio (10^5^ HUVEC seeded with 10^3^ ASC). A 1:0.01 seeding ratio was chosen to be able to observe even small changes of vasculogenic events whereas a 1:1 seeding ratio usually leads to a mature capillary network after one week even without stimulation. Therefore, it was possible to detect and quantify single-cell specific changes in morphology and proliferation (1:0.01 co-cultures) as well as influences of LED light therapy on the formation of mature networks.

### Quantification of fluorescent cells and vascular networks

In order to quantify the vascular structure formation in light-stimulated GFP-HUVEC/ASC fibrin matrices, images were taken on day 4 and 7 (4 images per clot) on a Leica DMI6000B epifluorescence microscope (Leica, Solms, Germany) in a resolution of 1392 × 1040 pixels and stored in a TIF format. These timepoints were chosen to evaluate early vasculogenic events (day 4) but also maturation of the capillary network formed (day 7). For quantifying cell morphology changes in co-culture ratios of 1:0.01 after 4 and 7 days of incubation, Cellprofiler software version 2.11 was used. Initially a color to gray conversion was performed to obtain grayscale images. Subsequently Cellprofiler’s object detection routine was used to detect the individual cells. No further processing of the images was required. The build in “global background” threshold routine with a threshold correction factor of 2 was used to obtain accurate detection of the cells. The total number of cells, the fraction of area covered with cells and the form factor of each cell was measured. The form factor is defined as 4 * π * area/perimeter^2^ (it equals 1 for a perfectly circular object) and represents cell elongation. The higher the form factor, the less elongated cells are. The obtained data was stored in comma separated values format and statistically analyzed.

### NO measurement

Supernatants of 1:0.01 HUVEC/ASC co-cultures were collected after 4 days of incubation and subjected to NO measurement. To assess the synthesis of nitric oxide (NO) after illumination, 2.4 × 10^4^ HUVECS were seeded in 24-well plates and grown to 80% confluence. The cells were illuminated and supernatant/medium samples were taken immediately and 4 days post-illumination. Nitric oxide was quantified with Sievers 280i-NO Analyzer (General Electrics) as previously described (Weidinger *et al*., Antioxid Redox Signal. 2015 Mar 1;22(7):572-86). Briefly, samples were injected into a glass vessel, containing vanadium chloride, a redox active reagent, which converts NO species into NO; this in turn reacts with ozone (O_3_) and gives a chemiluminescence signal.

### Protein expression

To determine protein expression changes mediated by LLLT in HUVEC:ASC co-cultures (1:0.01 ratio), supernatants of co-cultures were taken after 4 days of incubation and measured with an angiogenesis protein array kit (RnD systems, Minneapolis, USA) by investigating 55 different angiogenesis-related cytokines. Proteins which were ≥1.5 fold upregulated in light stimulated groups compared to control were considered. The protein blots were developed with an exposure time of 5 min.

### ROS formation

For measurement of hydrogen peroxide formation, HUVEC were seeded in four wells of four 24-well plates at a density of 2.5 * 104 cells/well. 24 hours later, at 70–80% confluence, cells were used for treatment. The medium was aspirated and replaced by 300 µL Krebs-HEPES buffer per well after two washing steps with 750 µL PBS/well. For inhibition of NADPH oxidase 3 µL of Diphenyleneiodonium (DPI – Sigma Aldrich, Germany, D2926), were added to two wells per plate to yield a final concentration of 1 µM. Each plate was then randomly assigned to one group and irradiated accordingly for 10 minutes at a distance of 2 cm, while the control plate was wrapped in tin foil. Samples were taken and measured 5 min, 15 min and 30 min after treatment.

For measurement 50 μL of the supernatant were transferred into two corresponding wells of a black, opaque 96-well plate (Greiner Bio-One GmbH, Austria) and 50 μL of Amplex® Red (Thermo Fisher Scientific Inc., Waltham, Massachusetts, A12222) horseradish peroxidase (HRP – Sigma Aldrich, Germany, P8250) solution was added. The color reaction was analyzed using a POLARstar® Omega plate reader (BMG LABTECH GmbH, Germany) by measuring the fluorescent emission at 590 nm (excitation wavelength at 544 nm).

### Statistics

Statistical differences were evaluated by using one-way ANOVA tests with Dunnett post testing to assess significances with prior evaluating the presence of Gaussian distributions. All data sets are presented as mean ± standard deviation. P-values less than 0.05 were considered as significant. All statistical analyses were performed with GraphPad Prism 4.0 software (GraphPad, San Diego, USA). All experiments were performed 6 times with HUVEC from two different single donors and one pooled donor HUVEC sample to reduce variation in combination with 3 diverging ASC donors for co-culture experiments. N = 6 for all experiments except the evaluation of protein expression after 4 days which was performed with pooled samples from three donors in duplicate.

## Electronic supplementary material

Supplementary Information

## References

[1] Rochkind S (2007). Efficacy of 780-nm Laser Phototherapy on Peripheral Nerve Regeneration after Neurotube Reconstruction Procedure (Double-Blind Randomized Study). Photomedicine and Laser Surgery.

[2] Aimbire F (2006). Low-Level Laser Therapy Induces Dose-Dependent Reduction of TNFα Levels in Acute Inflammation. Photomedicine and Laser Surgery.

[3] Silva Júnior AN (2002). Computerized Morphometric Assessment of the Effect of Low-Level Laser Therapy on Bone Repair: An Experimental Animal Study. Journal of Clinical Laser Medicine & Surgery.

[4] Tuby H, Maltz L, Oron U (2006). Modulations of VEGF and iNOS in the rat heart by low level laser therapy are associated with cardioprotection and enhanced angiogenesis. Lasers Surg. Med..

[5] Mittermayr R (2007). Blue Laser Light Increases Perfusion of a Skin Flap Via Release of Nitric Oxide from Hemoglobin. Mol Med.

[6] Dvorak HF, Brown LF, Detmar M, Dvorak AM (1995). Vascular permeability factor/vascular endothelial growth factor, microvascular hyperpermeability, and angiogenesis. Am J Pathol.

[7] Rohringer S (2014). Molecular and Cellular Effects of *In Vitro* Shockwave Treatment on Lymphatic Endothelial Cells. PLoS ONE.

[8] Dungel P (2014). Low level light therapy by LED of different wavelength induces angiogenesis and improves ischemic wound healing. Lasers Surg. Med..

[9] Park I-S, Chung P-S, Ahn JC (2015). Adipose-derived stromal cell cluster with light therapy enhance angiogenesis and skin wound healing in mice. Biochem. Biophys. Res. Commun..

[10] Colombo F (2013). Effect of low-level laser therapy (λ660 nm) on angiogenesis in wound healing: a immunohistochemical study in a rodent model. Braz Dent J.

[11] Ricci R, Pazos MC, Borges RE, Pacheco-Soares C (2009). Biomodulation with low-level laser radiation induces changes in endothelial cell actin filaments and cytoskeletal organization. Journal of Photochemistry and Photobiology B: Biology.

[12] Schindl A, Merwald H, Schindl L, Kaun C, Wojta J (2003). Direct stimulatory effect of low-intensity 670 nm laser irradiation on human endothelial cell proliferation. British Journal of Dermatology.

[13] Dungel P (2008). Illumination with blue light reactivates respiratory activity of mitochondria inhibited by nitric oxide, but not by glycerol trinitrate. Arch. Biochem. Biophys..

[14] Adamskaya N (2011). Light therapy by blue LED improves wound healing in an excision model in rats. Injury.

[15] Huang, Y.-Y., Sharma, S. K., Carroll, J. & Hamblin, M. R. Biphasic Dose Response in Low Level Light Therapy – an Update. *Dose-Response* **9**, dose–response. 11–009. Hamblin (2011). 10.2203/dose-response.11-009.Hamblin PMC3315174 22461763

[16] Barolet, D., Duplay, P., Jacomy, H. & Auclair, M. Importance of pulsing illumination parameters in low-level-light therapy. *J*. *Biomed*. *Opt* **15**, 048005–048005–8 (2010). 10.1117/1.3477186 20799848

[17] Brondon P, Stadler I, Lanzafame RJ (2009). Pulsing influences photoradiation outcomes in cell culture. Lasers Surg. Med..

[18] Holnthoner, W. *et al*. Adipose-derived stem cells induce vascular tube formation of outgrowth endothelial cells in a fibrin matrix. *Journal of Tissue Engineering and Regenerative Medicine* n/a–n/a, doi:10.1002/term.1620 (2012). 10.1002/term.1620 23038666

[19] Rohringer, S. *et al*. Mechanisms of vasculogenesis in 3D fibrin matrices mediated by the interaction of adipose-derived stem cells and endothelial cells. *Angiogenesis* 1–13, doi:10.1007/s10456-014-9439-0 (2014). 10.1007/s10456-014-9439-0 25086616

[20] Farivar S, Malekshahabi T, Shiari R (2014). Biological Effects of Low Level Laser Therapy. J Lasers Med Sci.

[21] Bossini PS (2008). Low-level laser therapy (670 nm) on viability of random skin flap in rats. Lasers Med Sci.

[22] Chen C-H, Hung H-S, Hsu S-H (2008). Low-energy laser irradiation increases endothelial cell proliferation, migration, and eNOS gene expression possibly via PI3K signal pathway. Lasers Surg Med.

[23] Szymanska J (2013). Phototherapy with low-level laser influences the proliferation of endothelial cells and vascular endothelial growth factor and transforming growth factor-beta secretion. J. Physiol. Pharmacol..

[24] Chaves ME, de A (2014). Effects of low-power light therapy on wound healing: LASER x LED. Anais Brasileiros de Dermatologia.

[25] Balzer JH (2015). Non-Thermal Dielectric Barrier Discharge (DBD) Effects on Proliferation and Differentiation of Human Fibroblasts Are Primary Mediated by Hydrogen Peroxide. PLoS One.

[26] Quent VM (2010). Discrepancies between metabolic activity and DNA content as tool to assess cell proliferation in cancer research. JCell Mol Med.

[27] Schuh CMAP (2014). *In vitro* extracorporeal shock wave treatment enhances stemness and preserves multipotency of rat and human adipose-derived stem cells. Cytotherapy.

[28] Teuschl A, Balmayor ER, Redl H, van Griensven M, Dungel P (2015). Phototherapy With LED Light Modulates Healing Processes in an *In Vitro* Scratch-Wound Model Using 3 Different Cell Types. Dermatologic Surgery.

[29] Fushimi T (2012). Green light emitting diodes accelerate wound healing: characterization of the effect and its molecular basis *in vitro* and *in vivo*. Wound Repair Regen.

[30] Ieda (2006). G-CSF and HGF: Combination of vasculogenesis and angiogenesis synergistically improves recovery in murine hind limb ischemia. YJMCC.

[31] Hedhli N (2012). Endothelial-derived neuregulin is an important mediator of ischaemia-induced angiogenesis and arteriogenesis. Cardiovasc Res..

[32] Kitlinska (2003). Dual Role of Dipeptidyl Peptidase IV (DPP IV) in Angiogenesis and Vascular Remodeling. Advances in Experimental Medicine and Biology.

[33] Ball KA (2011). Low intensity light stimulates nitrite-dependent nitric oxide synthesis but not oxygen consumption by cytochrome c oxidase: Implications for phototherapy. J Photochem Photobiol B.

[34] Plass CA (2012). Light-induced vasodilation of coronary arteries and its possible clinical implication. Ann Thorac Surg.

[35] Cury V (2013). Low level laser therapy increases angiogenesis in a model of ischemic skin flap in rats mediated by VEGF, HIF-1α and MMP-2. J. Photochem. Photobiol. B, Biol..

[36] Petzelbauer P, Bender JR, Wilson J, Pober JS (1993). Heterogeneity of dermal microvascular endothelial cell antigen expression and cytokine responsiveness *in situ* and in cell culture. J. Immunol..

[37] Wolbank S (2007). Dose-Dependent Immunomodulatory Effect of Human Stem Cells from Amniotic Membrane: A Comparison with Human Mesenchymal Stem Cells from Adipose Tissue. Tissue Engineering.

[38] Charwat V (2015). Potential and limitations of microscopy and Raman spectroscopy for live-cell analysis of 3D cell cultures. Jbiotec..

