# Supplementary material for: The impact of wavelengths of LED light-therapy on endothelial cells
Source: Sci Rep. 2017 Sep 6;7:10700. doi: 10.1038/s41598-017-11061-y (PMC5587748; doi:10.1038/s41598-017-11061-y)
Supplement: Supplementary file 1 — Supplementary Information [file 41598_2017_11061_MOESM1_ESM.doc]

**Supplementary information**

**The impact of wavelengths of LED light-therapy on endothelial cells**

Sabrina Rohringer^1,2,#^, Wolfgang Holnthoner^1,2^, Sidrah Chaudary^1,2^, Paul Slezak^1,2^, Eleni Priglinger^1,2^, Martin Strassl^3^, Karoline Pill^1,2^, Severin Mühleder^1,2^, Heinz Redl^1,2^, Peter Dungel^1,2^*

^1^ Ludwig Boltzmann Institute for Experimental and Clinical Traumatology, Donaueschingenstrasse 13, 1200 Vienna, Austria

^2^ Austrian Cluster for Tissue Regeneration, Vienna, Austria

^3^ Laser Consult Austria e.U., Salzburg, Austria

[*Peter.Dungel@trauma.lbg.ac.at](mailto:*Peter.Dungel@trauma.lbg.ac.at)

^#^ Current address: Max F. Perutz Laboratories, University of Vienna, Dr. Bohr-Gasse 9/3, 1030 Vienna, Austria

**Supplementary methods:**

**Plasmids and retroviral infection of ASC**

mCherry in pLV vectors and pBMN-Z were purchased from Addgene (Cambridge, USA). mCherry was subcloned into pBMN after digestion with *Bam*HI and *Sal*I. Phoenix ampho cells were a kind gift from Regina Grillari (University of Natural Resources and Life Sciences, Vienna) and cultured in DMEM 10% FCS. Virus particle generation was performed by transfecting Phoenix ampho cells at 80% confluency using lipofectamine 2000 or TurboFect (Thermo Fisher, Waltham, USA) according to the manufacturer’s instructions. Supernatant containing virus particles was mixed 50:50 with full medium and transferred onto 50% confluent ASC and incubated over night. mCherry-ASC were then expanded in new flasks and used for subsequent experiments.

**Immunofluorescence staining of tube-like structures in fibrin**

HUVEC/ASC co-cultures were cultured in 3D fibrin matrices prior to immunofluorescence staining. After one week, cells were fixed overnight on ice with 4% PFA. Cells were washed prior to incubation with mouse anti-human CD31-FITC antibody (BD Pharmingen) overnight on ice. To enhance fluorescence signal, cells were then additionally incubated with goat anti-mouse Alexa Fluor 488 secondary antibody (Thermo Fisher Scientific Inc.) overnight on ice. Fluorescence images were taken on a laser-scanning confocal microscope (Zeiss LSM510, Zeiss, Germany). Z-stack images were created and scale bars added to images using ImageJ.

**Matrigel assay**

To investigate the vasculogenic potential of HUVEC, cells were cultured on Matrigel, which is considered a standard assay to analyse vasculogenic capabilities. Matrigel Assay was performed in 24-well plates with 300µl Matrigel/well (BD Biosciences). After a matrix incubation period of 15 min at 37°C and 5% CO2, 2.5x10^5^ HUVEC, pooled from three individuals, resuspended in 500µl EGM-2 medium were added on top of the solidified matrix. After an incubation of 1h cells were illuminated with either red, green or blue LED for 10min. Tube formation was analysed after 24h by taking pictures and counting manually.

**Supplementary results:**


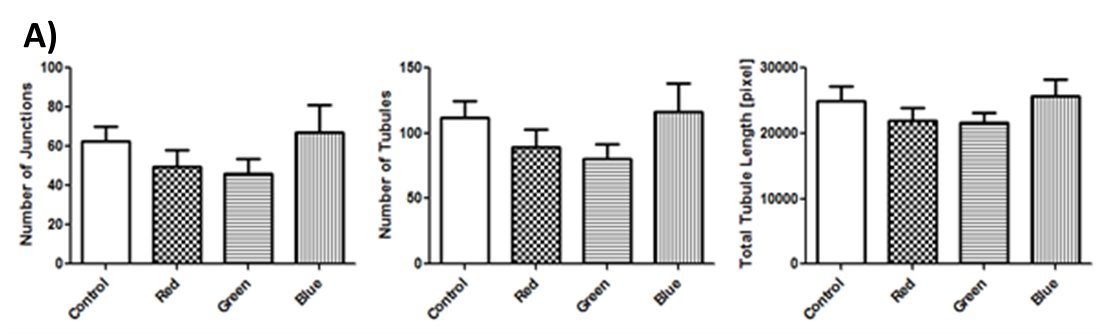


**Suppl. Figure 1.** Effects of LED light treatment by different wavelengths on tube formation in Matrigel assays. HUVEC were seeded on Matrigel, incubated for 1h and then illuminated with either wavelength at 50mW/cm^2^. Controls were left untreated. Tube formation was analysed 24h after treatment.

**
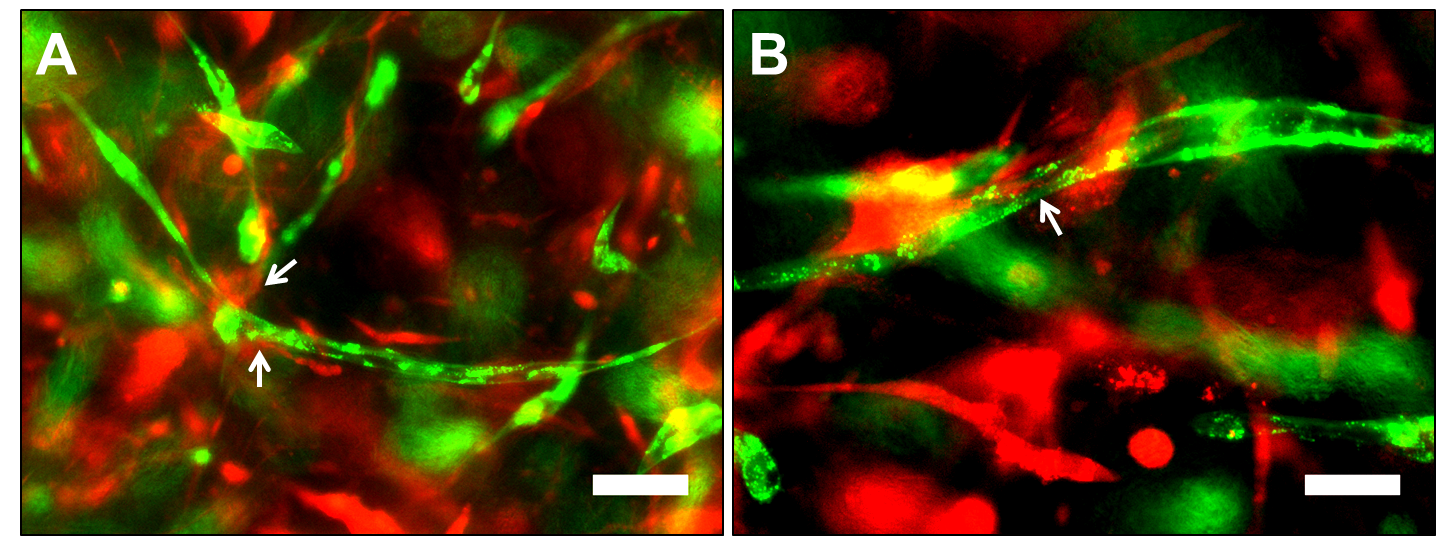
**

**Suppl. Figure 2:** Co-culture of mCherry-ASC with GFP-HUVEC in fibrin. (A,B) Representative imgages of fibrin clots having mCherry-ASC and GFP-HUVEC in a 1:1 ratio incorporated. After 1 week of incubation, red-fluorescent ASC were interacting with green endothelial microcapillaries (arrows). Scale bar: 100 µm (A) and 50 µm (B).

**
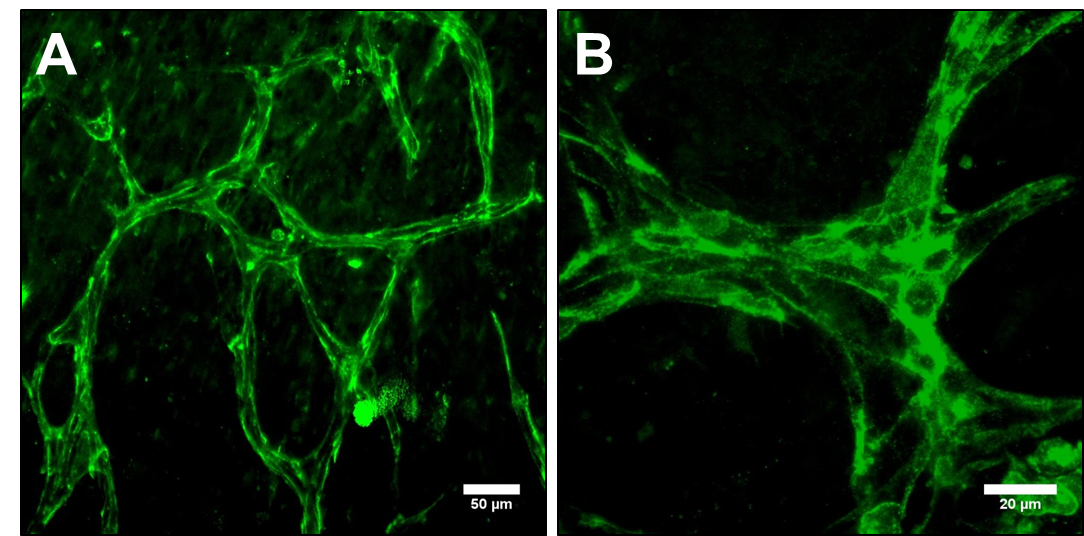
**

**Suppl. Figure 3:** Anti-CD31 immunofluorescence staining of HUVEC/ASC co-cultures in 3D fibrin matrices after one week of culture. Staining shows a clear localization of CD31 at the cell-cell-interfaces of tubular structures formed by HUVEC, indicating that functional adherens junctions have formed. Images show z-stacks obtained with a laser-scanning confocal microscope. Scale bar: 50 µm (A) and 20 µm (B).
